# Supplementary material for: Cell-to-cell expression dispersion of B-cell surface proteins is linked to genetic variants in humans
Source: Commun Biol. 2020 Jul 3;3:346. doi: 10.1038/s42003-020-1075-1 (PMC7335051; doi:10.1038/s42003-020-1075-1)
Supplement: Supplementary file 1 — Supplementary Information [file 42003_2020_1075_MOESM1_ESM.pdf]

Supplementary Information

Cell-to-cell expression dispersion of B-cell surface proteins is linked to genetic variants in humans

Gérard Triqueneaux[1,*], Claire Burny[1,*], Orsolya Symmons[1,*], Stéphane Janczarski[1], Henri Gruffat[2] and Gaël Yvert[1,#]

1) Laboratory of Biology and Modeling of the Cell, Ecole Normale Supérieure de Lyon, CNRS, Université Claude Bernard Lyon 1, Université de Lyon, 69007 Lyon, France.

2) CIRI-Centre International de Recherche en Infectiologie, Univ Lyon, Université Claude Bernard Lyon 1, Inserm, U1111, CNRS, UMR5308, ENS Lyon, 46 allée d'Italie, F-69007, Lyon, France.

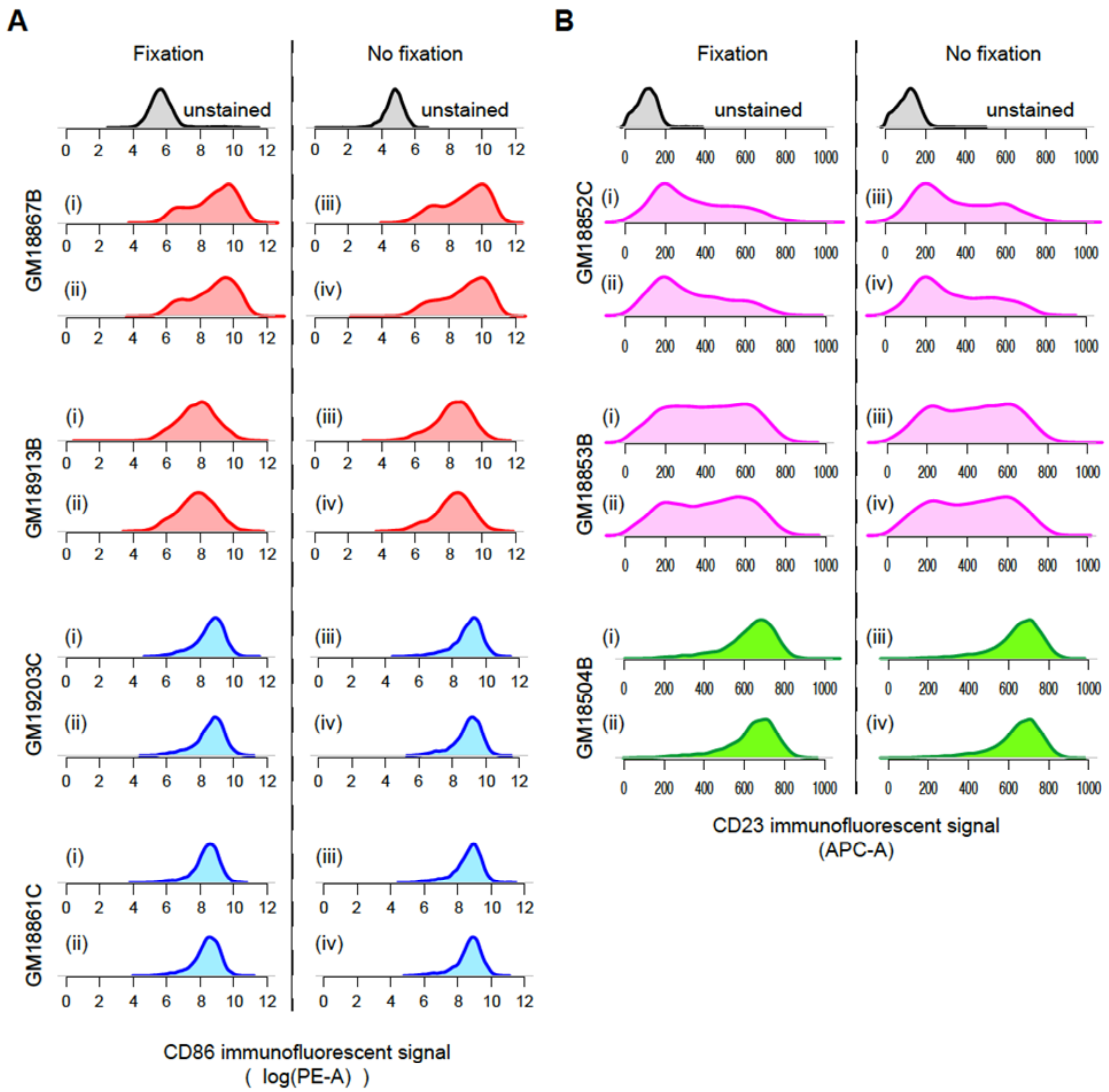

Supplementary Figure 1. Control of the effect of PFA-fixation on immunostaining. **A**) Cell lines described to have high (red) and low (blue) CD86 expression dispersion in the main study were re-cultured. Each culture was split in four samples. Samples i and ii were first fixed with PFA and then immunostained for CD86, as in the main study. Samples iii and iv were first immunostained, then washed and then fixed with PFA. The data shown correspond to cells that were gated on the basis of high FSC-A and low SSC-A values (to discard debris). Each gate was determined from the data using the curv2Filter::flowStats function[1] (package version 3.32.0). Each distribution shown is based on at least 2,500 gated cells. Grey: negative controls where the antibody was omitted. **B**) Cell lines described to be bimodal (magenta) or unimodal (green) for CD23 expression were re-cultured and processed as in A) using the anti-CD23 antibody.

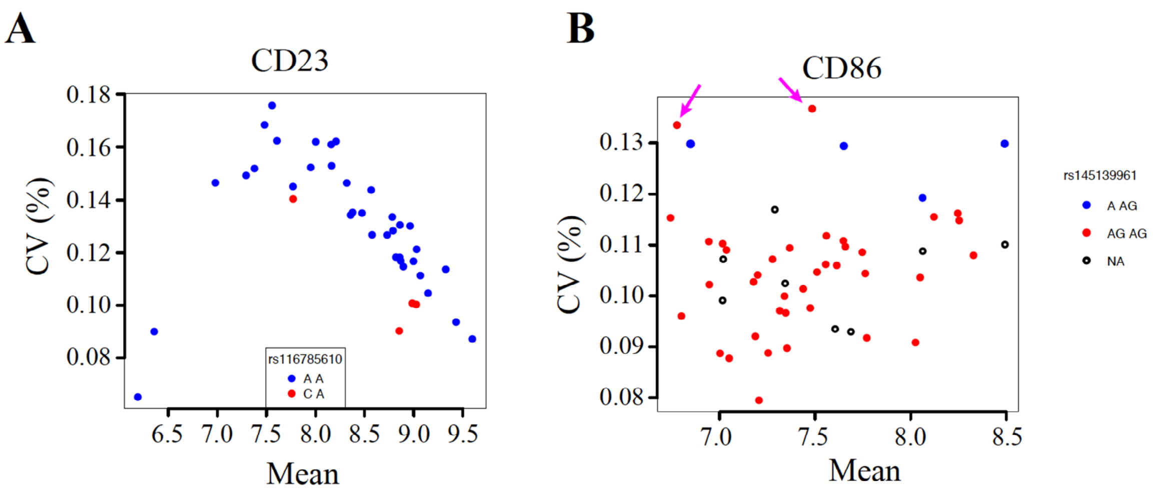

Supplementary Figure 2. A) Dot plot of CV vs. mean expression of CD23 in 40 LCLs, colored according to their genotype at SNP rs116785610. Association with expression dispersion was significant at $p = 10^{-4}$ nominally and at $p = 0.043$ when accounting for the number of SNPs tested. Note that linkage was searched for eight CD23 expression traits (mean, variability, dispersion and 5 traits describing bimodality, see methods). **B**) Dot plot of CV vs. mean expression of CD86, colored according to genotype at SNP rs145139961. NA: genotype not available. Arrows: Genotypes determined by PCR, rejecting association initially detected (Table 1).

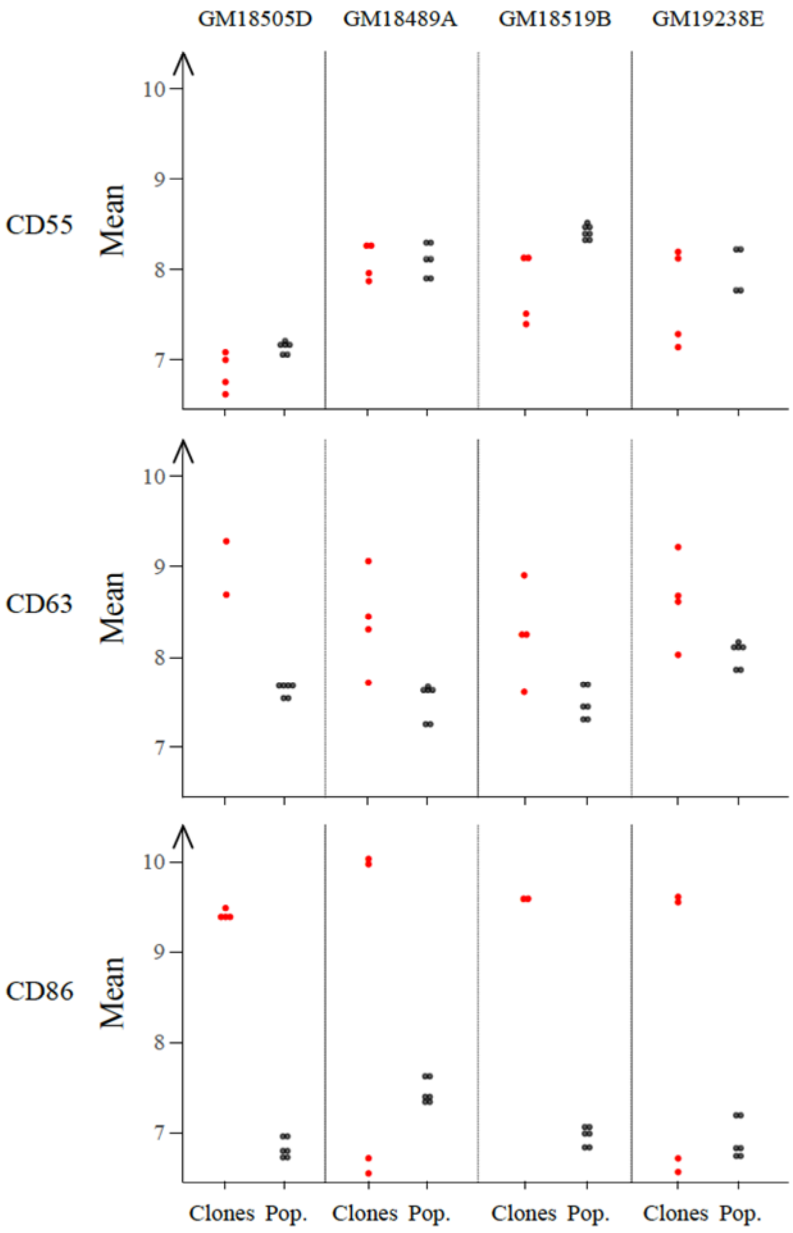

Supplementary Figure 3. Technical shift between two series of acquisitions. The survey of 50 LCLs (Pop., black dots) and the analysis of subclones (Clones, red dots) were performed in two distinct series of acquisitions, about 6 months apart. Four cell lines (indicated on top) were processed in both analyses. For CD63 and CD86, we observed a shift in mean values between the Pop. series and most samples of the Clones series, preventing reliable comparisons between the two series.

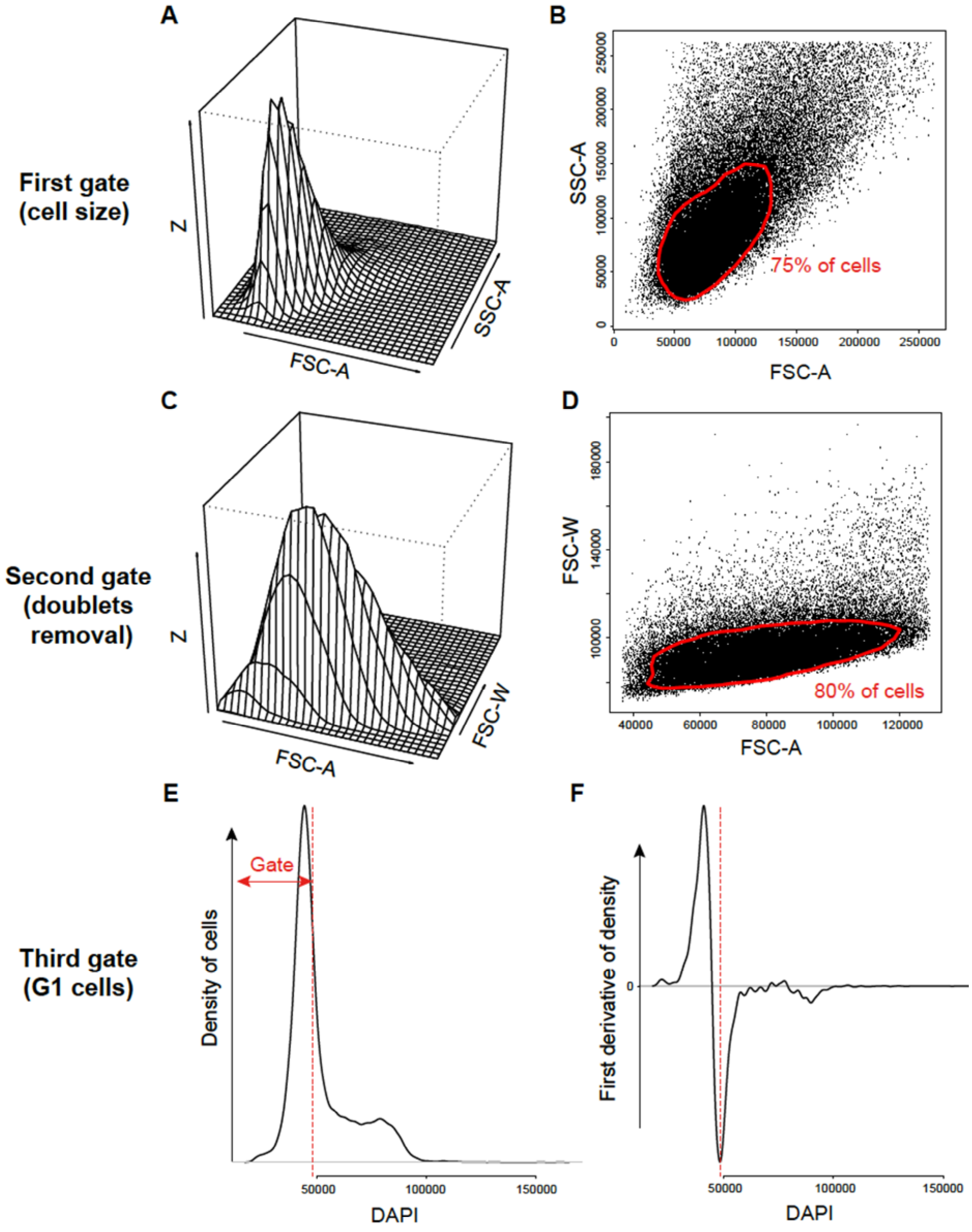

Supplementary Figure 4. Illustration of the gating strategy. For details, see the methods section of main text. Note that gates in A-D are defined after pooling cells from all samples. For clarity, only three samples were used to produce the figure. Z, density of cells.

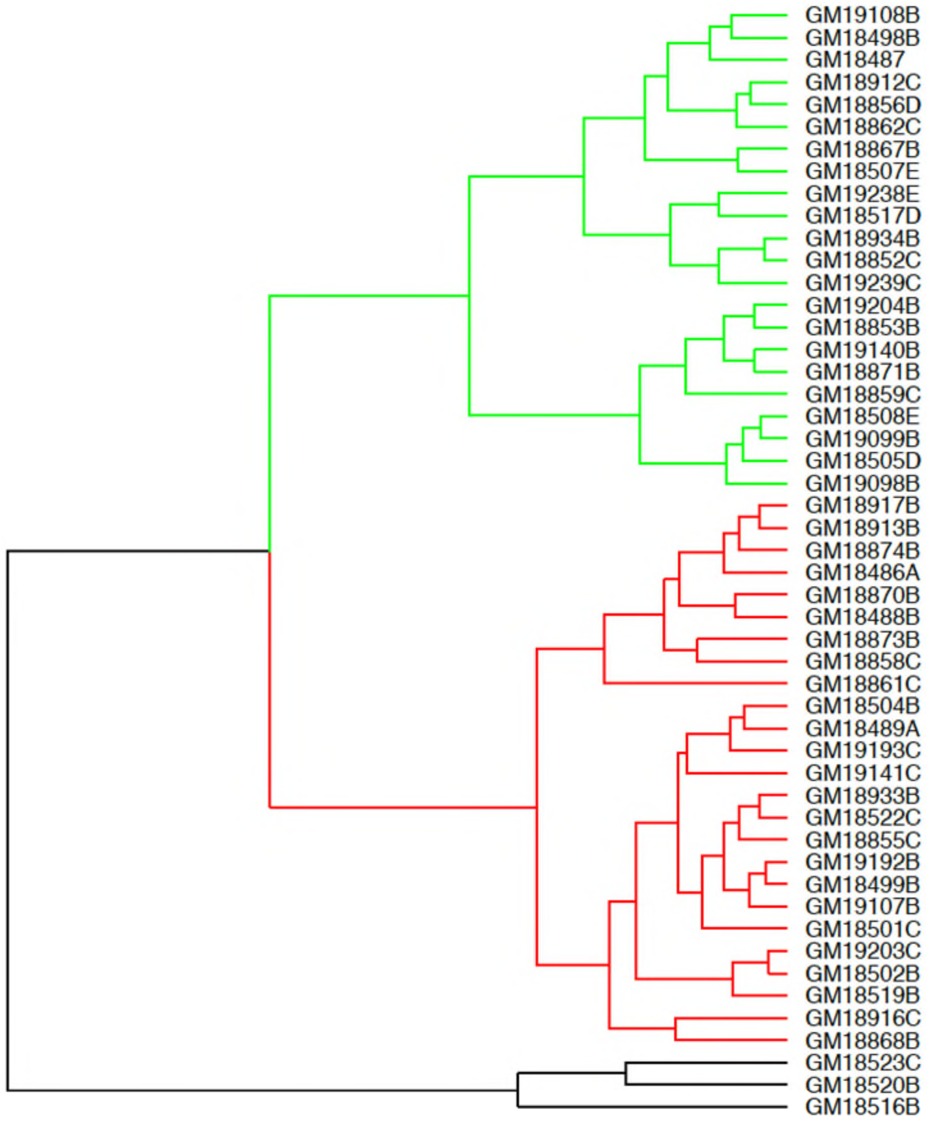

Supplementary Figure 5. Hierarchical clustering of cell lines based on CD23 GMM parameters, with names of the cell lines.

Supplementary Table 1: CDR3 genotypes obtained from clonality PCR. Numbers x/y are the percentages of counts corresponding to the CDR3 sequences indicated in columns, for samples of cell lines indicated in rows; x and y being the percentage in the first and second replicate sample of the cell line, respectively. Only sequences represented by at least 20% of the counts, based on at least 20 counts, are represented. (-) not detected in this sample; (i) sequence TSGNTGWYSDYWGQG corresponding to GM18505D cell line and subclones seems to have contaminated other samples; (ii) of a total of less than 100 counts; (*) of a total of more than 130,000 counts.

1A

	TSGNTGWYSDYWGQG (i)	**Conclusion**
GM18505D	85.7(*)/85.8(*)	monoclonal
5D-4F9	94.7(*)/94.9(*)	monoclonal
5D-5D10	95.5(*)/95.2(*)	monoclonal

1B

	AKHYDYWGQG	**Conclusion**
GM18486A	57.7(*)/52.5(*)	monoclonal (multiple other sequences were detected but did not reach 20% of representativity)
6A-1C10	95.8(*)	monoclonal
6A-2E9	95.3(*)/96.0(*)	monoclonal
6A-4F9	96.0(*)/96.2(*)	monoclonal
6A-5C3	97.0(*)/96.1(*)	monoclonal

1C

	ARGVMYDSGLISGWGQG	ARVGANNWYENDYWGQG	ARDLVAAAGTLGYWGQG	AKHYDYWGQG	TSGNTGWYSDYWGQG (i)	OTHERS	**Conclusion**
GM18519B	39.1/47					61.9/53	polyclonal
9B-3G7		80.5/95.6					monoclonal
9B-5B4		92.9/94.2					monoclonal
9B-5D4		84.8/92.4					monoclonal
9B-5E5		84.7/94.1					monoclonal
9B-2F4	-/44.0(ii)	94.0/ -					monoclonal
9B-4C3		93.9/81.5					monoclonal
9B-4G9		92.4/84.8					monoclonal
9B-5G5		94.0/86.3					monoclonal
9B-1D10			22.3	56.9			polyclonal
9B-2F10		91.9/ 66.5			-/26.0		monoclonal (i)
9B-2B5		91.5/55.5			-/39.3		monoclonal (i)

1D

	ARHHNYYDNSPEDWFDSWGHG	ARVGANNWYENDYWGQG	**Conclusion**
GM19239C	44.1/42.9(*)		unclear (one major sequence but not found in sequences from subclones)
9C-2F5		54.8(ii) / -	unclear (low coverage)
9C-6G5		50(ii)	unclear (low coverage)

1E

	VRDGGGWWYLYWGQG	AREEYSGSYYGYFQHWGQG	SRENDYSRSPEYWGQG	**Conclusion**
GM18489A	44.8/38.8	23.8/25.6		polyclonal
9A-3H10			92.3(*)/93.3(*)	monoclonal

1F

	ARDLVAAAGTLGYWGQG	ARDPAAGMQYFQHWGQG	AKVSAEFSTNWTFDFWGQG	AKAAMYYDSSGYMIFGGRWGQG	TSGNTGWYSDYWGQG (i)	AKHYDYWGQG	ARHHNYYDNSPEDWFDSWGHG	PAQYYFGYWGQGTLVTV	**Conclusion**
GM19238E	41.5/ 42.2	31.0/ 30.4							polyclonal
8E-3F7			89.4/ 90.3						monoclonal
8E-8E4				83.3/34.2	- / 59.1				monoclonal (i)
8E-2G6				89.6					monoclonal
8E-4G4				95.9/95.7					monoclonal
8E-6C10					31.7 (ii)/ 93.1 (ii)	26.8/ -	26.8/ -		polyclonal
8E-7E10					54.0 (ii) / 41.6 (ii)			27.0/ 37.7	unclear (i)
8E-1G6				25.2	71.2				unclear (i)

Supplementary Table 2: Primers used for CDR3 amplification and sequencing

ID	5'-3' sequence
1K47	TCGTCGGCAGCGTCAGATGTGTATAAGAGACAGTCTCCAAATGAACAGCCTGAGAGCC
1K48	TCGTCGGCAGCGTCAGATGTGTATAAGAGACAGGAGCTCTGTGACCGCCGCGGACACG
1K49	GTCTCGTGGGCTCGGAGATGTGTATAAGAGACAGACCTGAGGAGACGGTGACC
1K71	TCGTCGGCAGCGTCAGATGTGTATAAGAGACAGTGGAGCTGAGCAGCCTGAGATCTGA
1K72	TCGTCGGCAGCGTCAGATGTGTATAAGAGACAGCAATGACCAACATGGACCCTGTGGA
1K73	TCGTCGGCAGCGTCAGATGTGTATAAGAGACAGCAGCACCGCCTACCTGCAGTGGAGC
1K74	TCGTCGGCAGCGTCAGATGTGTATAAGAGACAGGTTCTCCCTGCAGCTGAACTCTGTG
1K75	TCGTCGGCAGCGTCAGATGTGTATAAGAGACAGCAGCACGGCATATCTGCAGATCAG

Supplementary Table 3. IgBLAST command lines used to extract CDR3 sequences.

ID	Command	Description
C1	makeblastdb -title human_gl_V -<br>parse_seqids -dbtype nucl -in<br>'"human_gl_VH_IgGermline.fasta"<br>"human_gl_VK_IgGermline.fasta"<br>"human_gl_VL_IgGermline.fasta"'<br>-input_type fasta -out<br>human_gl_V -hash_index	Create BLAST database of human V segments
C2	makeblastdb -title human_gl_D -<br>parse_seqids -dbtype nucl -in<br>'"human_gl_DH_IgGermline.fasta"'<br>-input_type fasta -out<br>human_gl_D -hash_index	Create BLAST database of human D segments
C3	makeblastdb -title human_gl_J -<br>parse_seqids -dbtype nucl -in<br>'"human_gl_JH_IgGermline.fasta"<br>"human_gl_JK_IgGermline.fasta"<br>"human_gl_JL_IgGermline.fasta"'<br>-input_type fasta -out<br>human_gl_J -hash_index	Create BLAST database of human J segments
C4	igblastn -query assembled.fasta<br>-germline_db_V human_gl_V -<br>germline_db_D human_gl_D -<br>germline_db_J human_gl_J -<br>auxiliary_data<br>./optional_file/human_gl.aux -<br>organism human -domain_system<br>imgt -outfmt 3 -show_translation<br>-num_alignments_V 1 -<br>num_alignments_D 1 -<br>num_alignments_J 1	Assign every read to one gene segment following the IMGT ontology (http://www.imgt.org)

Supplementary Table 4: List of antibodies used in this study

Human antigen	Host	Fluorescent Conjugate	Provider	Catalog Nb	Other
CD2	mouse	APC	BioLegend	300213	clone RPA-2.10, IgG1, κ
CD5	mouse	PE/Cy7	BioLegend	300622	clone UCHT2, IgG1, κ
CD9	mouse	FITC	BioLegend	312104	clone Hl9a, IgG1, κ
CD19	mouse	Alexa647	Invitrogen Molecular Probes	MHCD1921	clone SJ25-C1, IgG1
CD20	mouse	FITC	BioLegend	302304	clone 2H7, IgG2b, κ
CD22	mouse	APC	BioLegend	302510	clone HlB22, IgG1, κ
CD23	mouse	FITC	BD Pharmingen	561146	clone ML233, IgG1, κ
CD23	mouse	APC	BD BioSciences	N/A	clone ML233 at 100 μg/ml. Dilution used: 3.75/1000
CD37	mouse	FITC	BioLegend	356303	clone M-B371, IgG1, κ
CD38	mouse	PE/Cy7	BioLegend	303515	clone HlT2, IgG1, κ
CD40	mouse	PE/Cy7	BioLegend	334321	clone 5C3, IgG1, κ
CD46	mouse	FITC	BD Pharmingen	555949	clone E4.3, IgG2a, κ
CD53	mouse	PE	BioLegend	325406	clone Hl29, IgG1, κ
CD55	mouse	APC	BioLegend	311311	JS-11, IgG1, κ at 100 μg/ml. Dilution used: 3.75/1000
CD59	mouse	FITC	LifeTech/Invitrogen Molecular Probes	MHCD5901	clone MEM-43, IgG2a
CD63	mouse	PE/Cy7	BioLegend	353009	clone H5C6,

					IgG1, κ at 200 μg/ml. Dilution used: 4/1000
CD79b	mouse	PE	BD Pharmingen	557931	clone 3A2-2E7, IgG1, κ
CD80	mouse	Alexa647	BioLegend	305215	clone 2D10, IgG1, κ
CD86	mouse	PE	BioLegend	305405	IT2.2, IgG2b, κ at 100 μg/ml. Dilution used: 3.75/1000

Supplementary Table 5. Command lines used for linkage analysis.

ID	Command	Description
G1	`tabix -p vcf`	indexing VCF files (tabix v0.2.6)[2]
G2	`perl vcf-subset -c „40 LCLs IDs“ variants_chr.vcf.gz | bgzip -c > 40LCLs_variants_chr.vcf.gz`	subsetting VCF files for the set of 40 individuals with phased genotypes (vcftools v0.1.13)[3]
G3	`perl vcf-subset -c „8 LCLs IDs“ variants_chrall.vcf.gz | bgzip -c > 8LCLs_variants_chrall.vcf.gz`	subsetting VCF files for the set of 8 individuals with unphased genotypes (vcftools v0.1.13)[3].
G4	`perl vcf-merge 40LCLs_variants_chr.vcf.gz 8LCLs_variants_chrall.vcf.gz | bgzip -c > 48LCLs_variants_chr.vcf.gz`	Merging variants that are genotyped and uniquely identified in all 48 individuals (vcftools 0.1.13).
G5	`tabix -hp vcf chr.vcf.gz chr:begin-end | .bgzip -c > deltaTSS_chr.vcf.gz`	Select variants in regions flanking genes TSS.
G6	`vcftools --gzvcf deltaTSS_chr.vcf.gz –plink`	Convert VCF files into MAP and PED files
G7	`plink --file deltaTSS_chr --list-duplicate-vars`	removal of variants present in duplicates. (PLINK v1.9)[4]
G8	`plink –file deltaTSS_chr –exclude „IDs to remove“ --make-bed –out clean`	further removal of variants with non-unique IDs (duplicated IDs in second colmun of MAP file)
G9	`plink –bfile clean --recode –tab –out clean_nodup`	build final dataset
G10	`plink --bfile clean_nodup --maf`	Exclude variants with MAF < 0.05

	0.05 --hwe 0.001 --make-bed --out clean_nodup_filt	or deviating from Hardy-Weinberg.
G11	plink --bfile clean_nodup_filt --recode --tab --out clean_nodup_filt_tab	Re-encode data for PLINK after cleanup
L1	plink –bfile clean_nodup_filt_tab --assoc qt-means --mperm 10000 --adjust --out lm	Linkage test without dominance.
L2	plink –bfile clean_nodup_filt_tab --linear genotypic --mperm 10000 --out lm	Linkage test allowing dominance.

SUPPLEMENTARY REFERENCES

1. Hahne, F., Gopalakrishnan, N., Khodabakhshi, A., Wong, C. & Lee, K. *flowStats: Statistical methods for the analysis of flow cytometry data*. (2017).
2. Li, H. Tabix: fast retrieval of sequence features from generic TAB-delimited files. *Bioinformatics* **27**, 718–719 (2011).
3. Danecek, P. *et al.* The variant call format and VCFtools. *Bioinformatics* **27**, 2156–2158 (2011).
4. Chang, C. C. *et al.* Second-generation PLINK: rising to the challenge of larger and richer datasets. *GigaScience* **4**, 1–16 (2015).
