# Supplementary material for: Cell-to-cell expression dispersion of B-cell surface proteins is linked to genetic variants in humans
Source: Commun Biol. 2020 Jul 3;3:346. doi: 10.1038/s42003-020-1075-1 (PMC7335051; doi:10.1038/s42003-020-1075-1)
Supplement: Supplementary file 2 — Description of Additional Supplementary Files [file 42003_2020_1075_MOESM2_ESM.pdf]

Description of Additional Supplementary Files

File Name: Supplementary Data 1
Description: Expression traits summaries of each sample.

File Name: Supplementary Data 2
Description: Detailed results of the genetic linkage analysis (see included README file)

File Name: Supplementary Data 3
Description: Source data corresponding to main figures (see included README file)
