# Supplementary material for: Cell-to-cell expression dispersion of B-cell surface proteins is linked to genetic variants in humans
Source: Commun Biol. 2020 Jul 3;3:346. doi: 10.1038/s42003-020-1075-1 (PMC7335051; doi:10.1038/s42003-020-1075-1)
Supplement: Supplementary file 6 — Reporting Summary [file 42003_2020_1075_MOESM6_ESM.pdf]

nature research

Corresponding author(s): Gael YVERT

Last updated by author(s): Jun 4, 2020

Reporting Summary

Nature Research wishes to improve the reproducibility of the work that we publish. This form provides structure for consistency and transparency in reporting. For further information on Nature Research policies, see Authors & Referees and the Editorial Policy Checklist.

Statistics

For all statistical analyses, confirm that the following items are present in the figure legend, table legend, main text, or Methods section.

n/a	Confirmed	
☐	☒	The exact sample size (*n*) for each experimental group/condition, given as a discrete number and unit of measurement
☐	☒	A statement on whether measurements were taken from distinct samples or whether the same sample was measured repeatedly
☐	☒	The statistical test(s) used AND whether they are one- or two-sided<br>*Only common tests should be described solely by name; describe more complex techniques in the Methods section.*
☒	☐	A description of all covariates tested
☐	☒	A description of any assumptions or corrections, such as tests of normality and adjustment for multiple comparisons
☐	☒	A full description of the statistical parameters including central tendency (e.g. means) or other basic estimates (e.g. regression coefficient) AND variation (e.g. standard deviation) or associated estimates of uncertainty (e.g. confidence intervals)
☐	☒	For null hypothesis testing, the test statistic (e.g. *F*, *t*, *r*) with confidence intervals, effect sizes, degrees of freedom and *P* value noted<br>*Give P values as exact values whenever suitable.*
☒	☐	For Bayesian analysis, information on the choice of priors and Markov chain Monte Carlo settings
☒	☐	For hierarchical and complex designs, identification of the appropriate level for tests and full reporting of outcomes
☒	☐	Estimates of effect sizes (e.g. Cohen's *d*, Pearson's *r*), indicating how they were calculated

Our web collection on statistics for biologists contains articles on many of the points above.

Software and code

Policy information about availability of computer code

Data collection	Data collection is detailed in Methods section of the manuscript; our analysis code is made available.
Data analysis	Data analysis is detailed in Methods section of the manuscript, including the third-party software codes that were used; our analysis code is made available at https://gitbio.ens-lyon.fr/LBMC/gylab/lcl/ under the open source CeCill licence, as indicated in Code Availability section.

For manuscripts utilizing custom algorithms or software that are central to the research but not yet described in published literature, software must be made available to editors/reviewers. We strongly encourage code deposition in a community repository (e.g. GitHub). See the Nature Research guidelines for submitting code & software for further information.

Data

Policy information about availability of data

All manuscripts must include a data availability statement. This statement should provide the following information, where applicable:

- Accession codes, unique identifiers, or web links for publicly available datasets
- A list of figures that have associated raw data
- A description of any restrictions on data availability

As mentioned in the Data availability section, data was deposited at EBI: (https://www.ebi.ac.uk/ena/) under accession number PRJEB37875 and (http://www.ebi.ac.uk/biostudies) under accession number S-BSST382

Field-specific reporting

Please select the one below that is the best fit for your research. If you are not sure, read the appropriate sections before making your selection.

☒ Life sciences ☐ Behavioural & social sciences ☐ Ecological, evolutionary & environmental sciences

For a reference copy of the document with all sections, see nature.com/documents/nr-reporting-summary-flat.pdf

Life sciences study design

All studies must disclose on these points even when the disclosure is negative.

Sample size	The study includes numerous statistical considerations which are all detailed in the Methods section of the manuscript
Data exclusions	The study includes numerous statistical considerations which are all detailed in the Methods section of the manuscript
Replication	The study includes numerous statistical considerations which are all detailed in the Methods section of the manuscript
Randomization	The study includes numerous statistical considerations which are all detailed in the Methods section of the manuscript
Blinding	Blinding was not necessary: our study covered the extent of variation between samples and individuals. It did not assign specific observations to specific samples or individuals.

Reporting for specific materials, systems and methods

We require information from authors about some types of materials, experimental systems and methods used in many studies. Here, indicate whether each material, system or method listed is relevant to your study. If you are not sure if a list item applies to your research, read the appropriate section before selecting a response.

Materials & experimental systems

n/a	Involved in the study	
☐	☒	Antibodies
☐	☒	Eukaryotic cell lines
☒	☐	Palaeontology
☒	☐	Animals and other organisms
☒	☐	Human research participants
☒	☐	Clinical data

Methods

n/a	Involved in the study	
☒	☐	ChIP-seq
☐	☒	Flow cytometry
☒	☐	MRI-based neuroimaging

Antibodies

Antibodies used	Antibodies references and suppliers are listed in Table S4
Validation	we did not perform validation tests except to rule out fixation artifacts (see text).

Eukaryotic cell lines

Policy information about cell lines

Cell line source(s)	We used cell lines from the Coriell Institute, as described in Methods.
Authentication	none of the cell lines used were authenticated
Mycoplasma contamination	Cell lines were not tested for Mycoplasma contamination
Commonly misidentified lines (See ICLAC register)	none

Flow Cytometry

Plots

Confirm that:

☒ The axis labels state the marker and fluorochrome used (e.g. CD4-FITC).

☒ The axis scales are clearly visible. Include numbers along axes only for bottom left plot of group (a 'group' is an analysis of identical markers).

☒ All plots are contour plots with outliers or pseudocolor plots.

☒ A numerical value for number of cells or percentage (with statistics) is provided.

Methodology

Sample preparation	detailed in Methods section of the manuscript
Instrument	FacsCantoII from BD Biosciences
Software	See Methods and custom code made available.
Cell population abundance	no sorting
Gating strategy	dynamic gating is described in methods and illustrated in Supplementary Fig. 4

☒ Tick this box to confirm that a figure exemplifying the gating strategy is provided in the Supplementary Information.
